# ERA5-based global meteorological wildfire danger maps

**DOI:** 10.1038/s41597-020-0554-z

**Published:** 2020-07-07

**Authors:** Claudia Vitolo, Francesca Di Giuseppe, Christopher Barnard, Ruth Coughlan, Jesus San-Miguel-Ayanz, Giorgio Libertá, Blazej Krzeminski

**Affiliations:** 1 European Centre for Medium-range Weather Forecasts, Reading, United Kingdom; 2 European Commission, Joint Research Centre, Ispra, Italy

**Keywords:** Environmental impact, Natural hazards

## Abstract

Forest fires are an integral part of the natural Earth system dynamics, however they are becoming more devastating and less predictable as anthropogenic climate change exacerbates their impacts. In order to advance fire science, fire danger reanalysis products can be used as proxy for fire weather observations with the advantage of being homogeneously distributed both in space and time. This manuscript describes a reanalysis dataset of fire danger indices based on the Canadian Fire Weather Index system and the ECMWF ERA5 reanalysis dataset, which supersedes the previous dataset based on ERA-Interim. The new fire danger reanalysis dataset provides a number of benefits compared to the one based on ERA-Interim: it relies on better estimates of precipitation, evaporation and soil moisture, it is available in a deterministic form as well as a probabilistic ensemble and it is characterised by a considerably higher spatial resolution. It is a valuable resource for forestry agencies and scientists in the field of wildfire danger modeling and beyond. The global dataset is produced by ECMWF, as the computational centre of the European Forest Fire information System (EFFIS) of the Copernicus Emergency Management Service, and it is made available free of charge through the Climate Data Store.

## Abstract (summary)

**Table:** 

Measurement(s)	wetness of soil • wildfire • effort required for fire suppression • fire intensity
Technology Type(s)	computational modeling technique
Sample Characteristic - Environment	forest ecosystem
Sample Characteristic - Location	Earth (planet)

Machine-accessible metadata file describing the reported data: 10.6084/m9.figshare.12497732

## Background & Summary

Forest fires are an integral part of the natural Earth system dynamics, however they are becoming more devastating and less predictable as anthropogenic climate change exacerbates their impacts^1^. Longer fire seasons, more extreme fire weather and more rain-free days globally are inducing significant variations in wildfire danger^2^. Indeed, the fire community would benefit from more data on fire observations^3^ to characterise temporal trends as well as identify and quantify impacts on population, ecosystems and infrastructures. However, fire observations present a number of limitations. On the one hand, event catalogues are collated by local authorities. As such, they are often only available in the local language and use different standards for data collection making the information fragmented and unevenly distributed over the globe. The Joint Research Centre Data Catalogue (https://data.jrc.ec.europa.eu/) tries to overcome this issue by collecting, in a single data portal, information about wildfires in Europe (amongst other natural or man-made disasters). The catalogue is linked to the Copernicus Emergency Management Service, which provides timely geospatial information in support of emergency management activities following a disaster. This is an on-demand service, available since 2014, that can only be activated by authorised users and relies on limited resources (there is a limit on the number of multiple simultaneous activations that can be processed). As such, it cannot be used to derive long term statistics.

Remotely sensed observations, on the other hand, have been available for decades but fire activities have only been reliably monitored since early 2000. While one can observe fire occurrences, these occurrences are difficult to predict as the large majority of events are ignited by humans due to negligence or arson. For this reason, fire danger models do not predict where an ignition is likely to occur but rather the meteorological conditions that would cause flames to spread out of control, conditional to an occurred ignition^4^. These conditions, called ‘fire weather’, depend on atmospheric variables such as accumulated precipitation, relative humidity, temperature and wind speed. As such, fire weather related danger indices quantify a potential danger, not a real one.

In this context, historical fire weather can be assessed calculating fire danger indices from weather variables measured at monitoring stations. These are available at discrete locations, regions characterised by low density of monitoring stations consequently suffer from unreliable fire weather estimations. An alternative is to use reanalysis datasets, which provide a more spatially and temporally homogeneous alternative to point-based observations. A reanalysis dataset is a retrospective analysis where a numerical weather prediction model is used to generate a first-guess estimate of the past state of the climate which is then updated using observations^5,6^. Although errors and uncertainties of the reanalysis process are only partially known, these datasets are very often used as proxy for observations^7^. Reanalysis data also span multiple decades, which makes them the ideal resource to investigate long-term statistics and trends.

The European Centre for Medium-range Weather Forecasts (ECMWF) has recently developed an open source fire danger model, called the Global ECMWF Fire Forecast model (GEFF, currently at version 3.1, https://git.ecmwf.int/projects/CEMSF/repos/geff)^4^. GEFF implements, among others danger metrics, the Canadian Fire Weather Index (FWI) system^8,9^. The FWI system is able to model fuel moisture response to atmospheric forcing. Three fuel beds are considered at increasing depths. The deeper and more compact the fuel is, the slower its response to the atmospheric forcings. These moisture codes are then combined to derive fire behavior indices in terms of rate of spread and intensity. The FWI system is used to estimate fire danger in Europe and worldwide^10,11^ and it is an important component of the European Forest Fire Information System (EFFIS) as well as the Global Wildfire Information System (GWIS, https://gwis.jrc.ec.europa.eu/). The latter is a joint initiative of the GEO (http://www.earthobservations.org) and the Copernicus Work Programs (https://www.copernicus.eu/en/services/emergency). Using GEFF, ECMWF computes fire danger indices in both reanalysis and forecast mode (with a 10-day time horizon). Daily fire forecasts feed into the EFFIS and GWIS web viewers, while the reanalysis is part of the open data catalogue of the Copernicus Climate Data Store (CDS, https://cds.climate.copernicus.eu).

This paper describes GEFF-ERA5, the reanalysis dataset of FWI fire behavior indices based on the ERA5 reanalysis, which supersedes the previous fire danger reanalysis based on ERA-Interim (GEFF-ERAI^12^). The new reanalysis provides better precipitation, evaporation and soil moisture estimates and allows to calculate fire danger indices at much higher spatial resolution compared to ERA-Interim^13^. Unlike its predecessor, GEFF-ERA5 is made available as a deterministic as well as probabilistic model output, the latter allows to quantify uncertainties in the estimations which was not possible previously.

This dataset is a valuable resource for forestry agencies and scientists in the field of wildfire danger modeling and beyond. As such, the Copernicus programme is committed to release these layers free of charge now and in the future.

## Methods

In early 2019, ECMWF released a new generation reanalysis database, ERA5^13,14,15^, whose main variables, temperature, humidity, precipitation and wind speed are used to drive the GEFF model. Atmospheric fields from ERA5 undergo some pre-processing to represent atmospheric conditions at a nominal 12:00 local noon when fire conditions are considered to be at their worst in most regions. Like any weather prediction model, ERA5 integration at any time step simulates the atmospheric conditions at the same UTC time. This will correspond to different local times depending on the location. To produce a snapshot at 12:00 noon local time everywhere in the word, a temporal collage of the first 24-h forecast is performed. Thus atmospheric fields (e.g. temperature) are cut into 1-hourly time strips using the closest forecast step and then concatenated together so that the final field is representative of the conditions around the local noon within the 1-h resolution available. Using this method, the driving forcings are a composite of forecast outputs at different lead times in a 24-h interval and allows GEFF to provide globally coherent maps of fire danger indices^4^. The data pre-processing and model calculation is performed using a job scheduler software developed at ECMWF (ecFlow) which allows the executions of several tasks in parallel following strict scheduling and triggering rules. Despite ECMWF’s extensive computer resources the calculation of the 1979–2019 database takes around 10 days to complete. With 80% of the time being used to prepare the atmospheric fields. All outputs are uploaded directly to the climate data store as NetCDF files and also backed up on ECMWF servers.

The GEFF-ERA5 fire danger reanalysis dataset^16^ is made of four types of products: (i) deterministic model outputs (called simply’reanalysis’ on the CDS), (ii) probabilistic model outputs (made of 10 ensemble members), (iii) ensemble mean and (iv) ensemble spread. Probabilistic model outputs allow to calculate uncertainties associated with model estimates and are an important addition compared to the previous dataset based on ERA-Interim, which was completely deterministic. Each product contains seven gridded fire danger indices, archived monthly on the CDS. At the time of writing, data are available from January 1979 to December 2019 (this is also called ‘Consolidated dataset’ on the CDS). As the process of consolidating and releasing the official dataset takes about two months, the CDS also hosts a so called ‘Intermediate dataset’. This is an experimental dataset published 5 days behind real time that could be affected by changes at any point before the official release date. In future releases, the consolidated dataset will cover the period January 1950 to near real time.

Data are made available in NetCDF format with a daily time step at 12 noon local time on a regular unprojected grid with spherical coordinates expressed in decimal degrees (EPSG:4326). Latitudes span the range from −90 to +90 degrees and are referenced to the equator. Longitudes are in the range from 0 to 360 degrees, referenced to the Greenwich Prime Meridian, consistently with ERA5 and derived products. The spatial resolutions is 0.25° × 0.25° (about 28 km grid cell size) for the deterministic model, and 0.50° × 0.50° (about 56 km) for the ensemble members, mean and spread. This reanalysis dataset is obtained as a single model run starting on 1st January 1979. However, the first year is discarded to ignore the effects of the initial model spin-up.

**Fig. 1:**
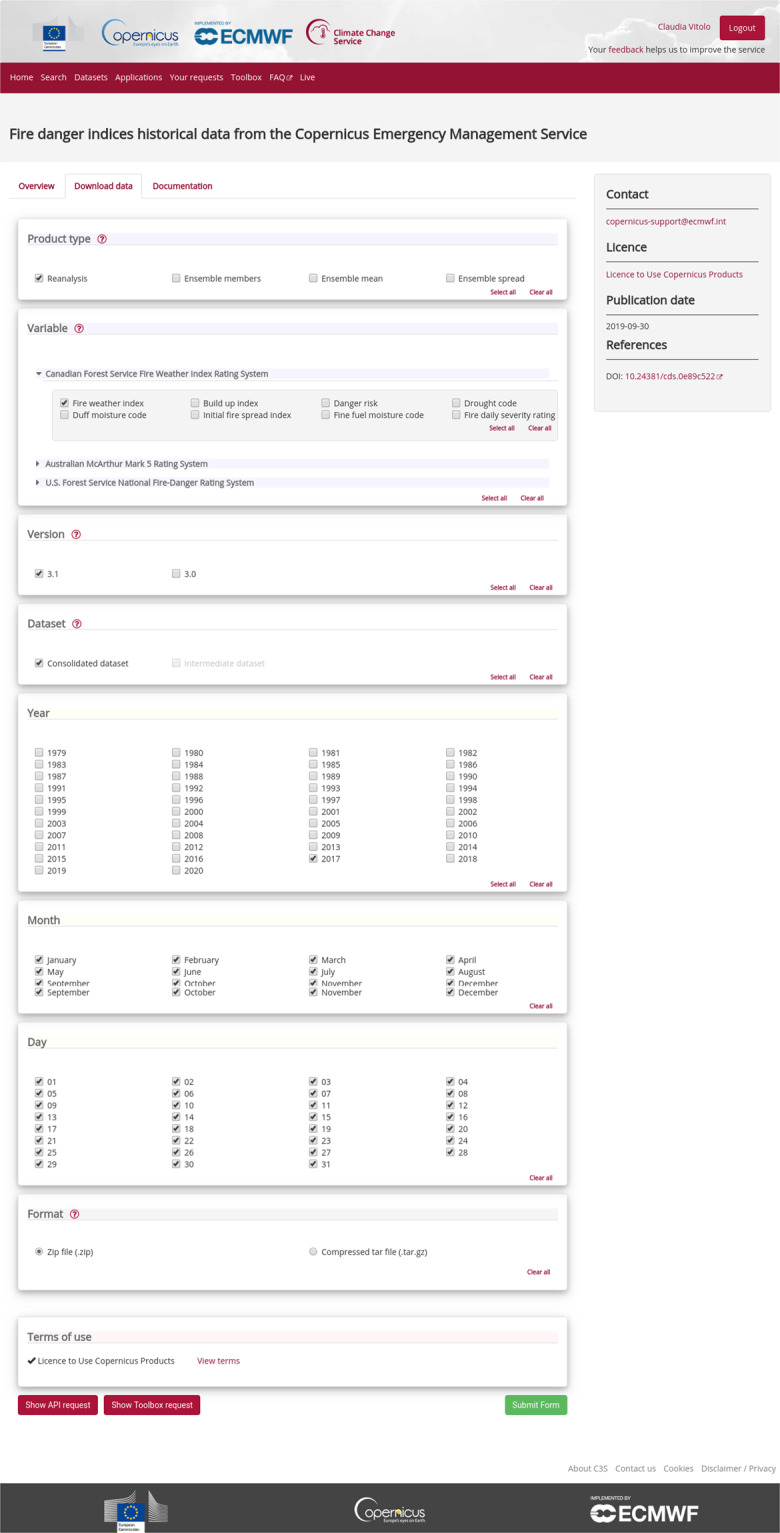
Copernicus Climate Data Store. Web interface to download historical simulations of fire danger indices from the Copernicus Emergency Management Service.

Data can be obtained from the CDS catalogue. Users need to register an account to download data via the web interface (Fig. 1). For batch download and programmatic access, users are encouraged to use the CDS API (https://cds.climate.copernicus.eu/api-how-to) and related python client which can be installed via the package management system *pip*. As an example, once the client is installed, the FWI index for the year 2017 can be retrieved using the script below.

**Fig. 2:**
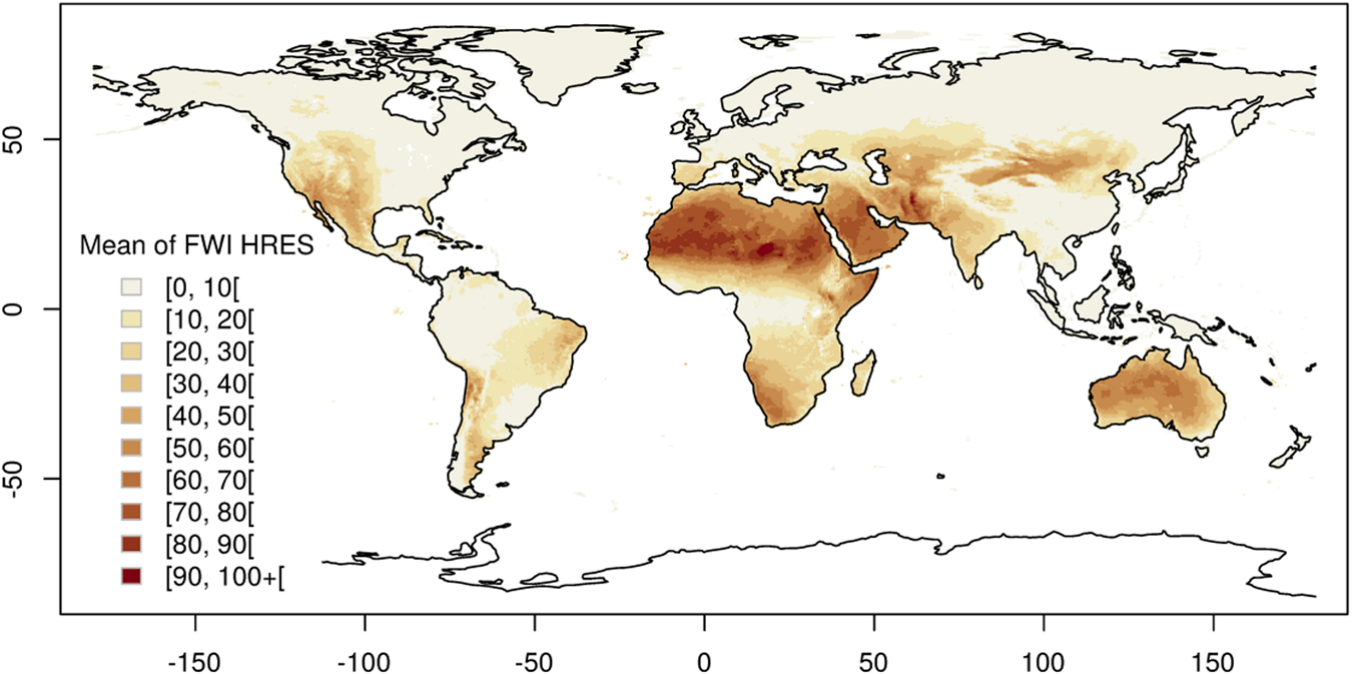
FWI HRES. Spatial distribution of the mean FWI (unmasked) values from the high resolution (HRES) model in 2017.

**Fig. 3:**
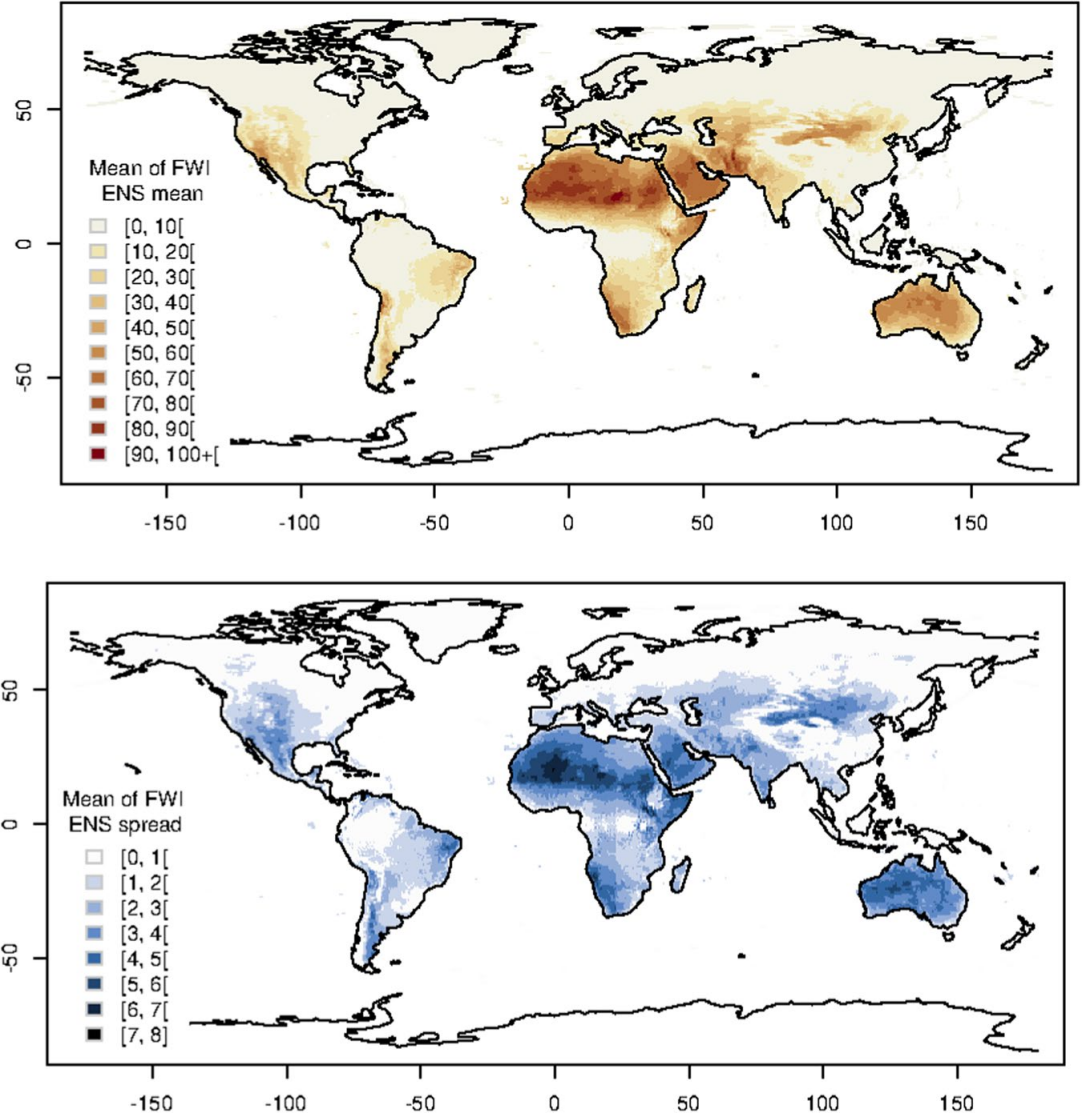
FWI ENS. Spatial distribution of the mean (top panel) and spread (bottom panel) FWI (unmasked) values from the ensemble (ENS) model in 2017.

**Fig. 4:**
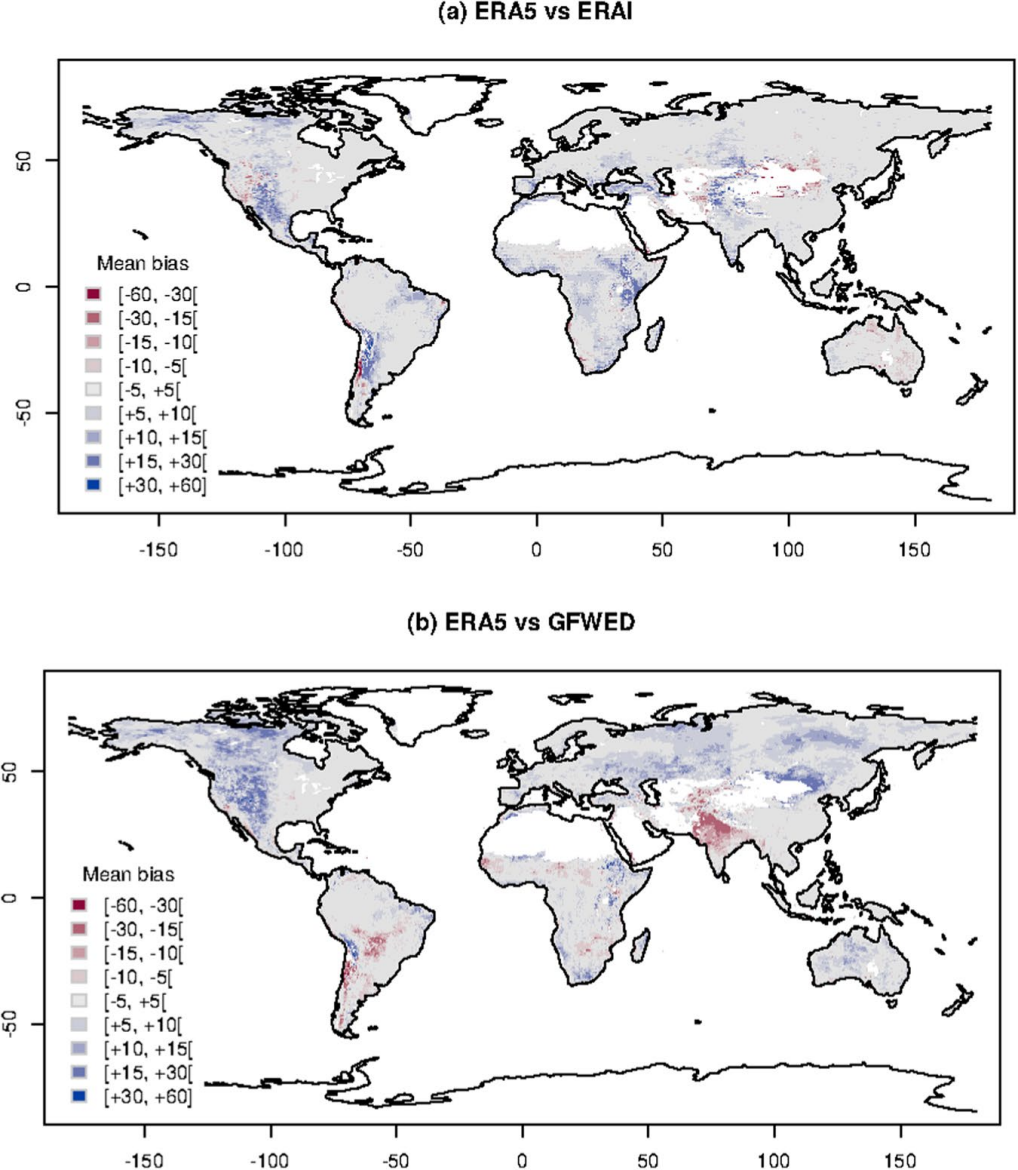
Mean bias. Spatial distribution of mean bias of FWI between GEFF-ERA5 and (top panel) GFWED or (bottom panel) GEFF-ERAI.

**Fig. 5:**
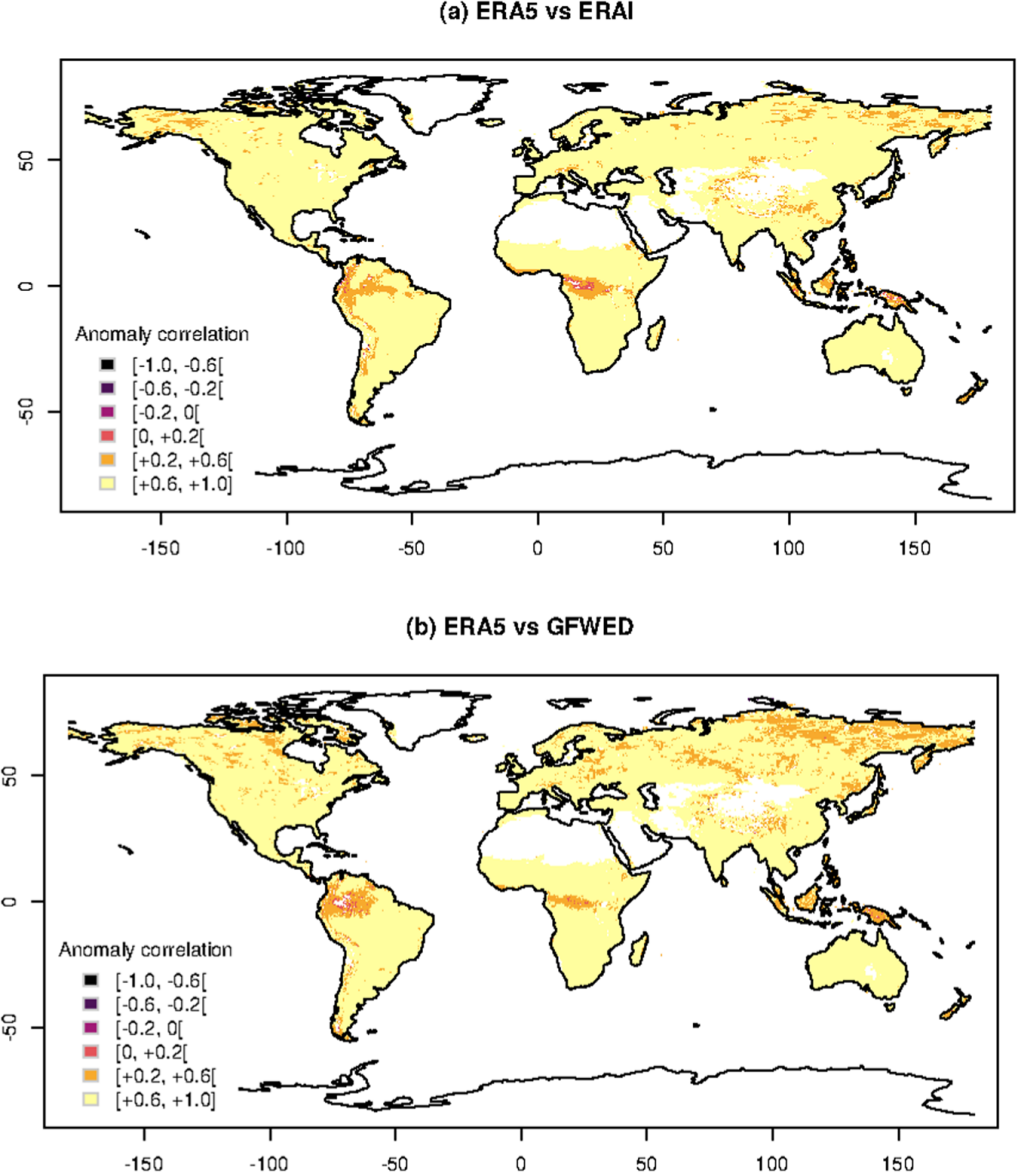
Anomaly correlation. Spatial distribution of anomaly correlation of FWI between GEFF-ERA5 and (top panel) GFWED or (bottom panel) GEFF-ERAI.

**Fig. 6:**
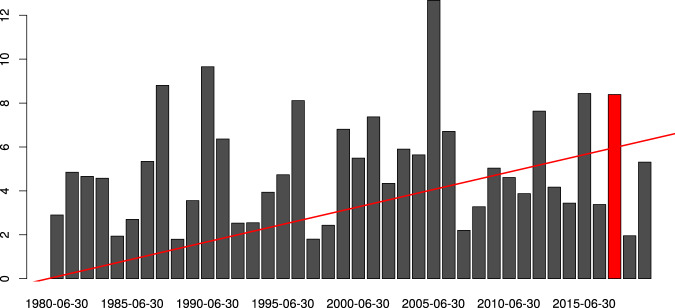
Monthly spread. The monthly spread of the ensemble at Pedrógão Grande (Portugal) is compared to the linear daily trend over 40 years of ERA5 reanalysis. The red bar highlights the spread in June 2017.

**Fig. 7:**
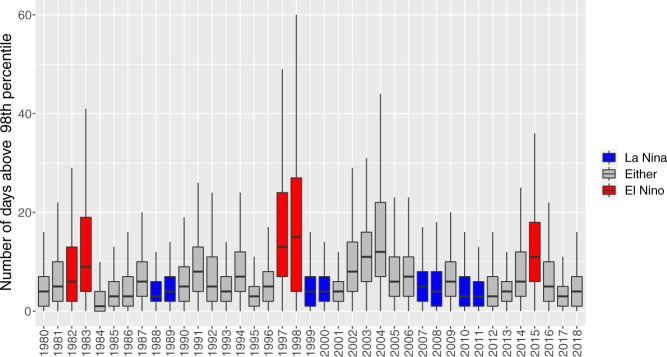
Number of days in South East Asia (see extent in Fig. 8) in which the FWI was above its 98^th^ percentile. For every grid cell and day of the year the climatology is calculated as average of the recorded FWI for all the years in the data record (1980–2018) and across a moving window of 9 days centered on the given day.

**Fig. 8:**
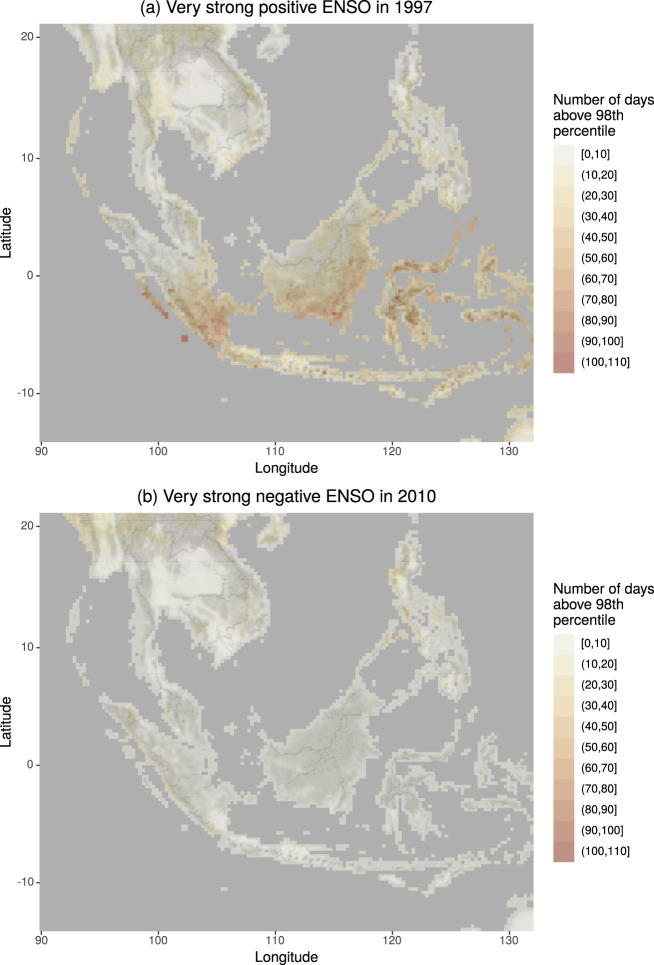
Map of days above the 98^th^ percentile. Number of days in which the FWI is above the 98^th^ percentile of its climatological distribution during the very strong positive ENSO in 1997 (top panel) and the very strong negative ENSO in 2010 (bottom panel).

import cdsapi

c = cdsapi.Client()

c.retrieve(

             'cems-fire-historical',

             {

                          'format':'zip',

                          'product_type':'reanalysis',

                          'variable':'fire_weather_index',

                          'version':'3.1',

                          'dataset':'Consolidated dataset',

                          'year':'2017',

                          'month':[

                                       '01','02','03',

                                       '04','05','06',

                                       '07','08','09',

                                       '10','11','12'

                          ],

                          'day':[

                                       '01','02','03',

                                       '04','05','06',

                                       '07','08','09',

                                       '10','11','12',

                                       '13','14','15',

                                       '16','17','18',

                                       '19','20','21',

                                       '22','23','24',

                                       '25','26','27',

                                       '28','29','30',

                                       '31'

                          ]

             },

             'download.zip')

## Data Records

The Canadian FWI system consists of seven indices: three measures of soil moisture, three fire behavior indices and one index related to ease of fire suppression. In the CDS, fire danger indices based on the Canadian FWI system as well as other systems (Australian McArthur Mark 5 Rating System^17,18^ and U.S. Forest Service National Fire-Danger Rating System^19^) are available^16^. However, the implementation of the Mark 5 and NFDRS systems are considered experimental and therefore they are not described in this manuscript.

The FWI dataset contains three measures of soil moisture calculated with different codes depending on the fuel type and layer depth:

- The **Fine Fuel Moisture Code** (FFMC), refers to the moisture content of litter and other cured fine fuels occupying the first fuel bed layers (surface layer, 1–2 cm deep). This code is an indicator of the relative ease of ignition and the flammability of fine fuel and it is characterised by a fast response to weather variations, with a timelag of 16 hours under standard conditions. FFMC is a unit-less number defined in the range [2, 101], with a conventional starting value of 85. In the CDS, this variable is called *fine_fuel_moisture_code*.
- The **Duff Moisture Code** (DMC), refers to the moisture content of loosely compacted organic layers of moderate depth (duff layer, 5–10 cm) and gives an indication of fuel consumption in moderate duff layers and medium-size woody material. DMC is characterised by a medium-term response (about 10–12 days) to weather variations. This is also a numeric rating but defined in the range [0, +∞], with a default startup value of 6. In the CDS, this variable is called *duff_moisture_code*.
- The **Drought Code** (DC), refers to the moisture content of deep, compact organic layers (deep duff layer, 10–20 cm). This code is a useful indicator of seasonal drought effects on forest fuels and the amount of smoldering in deep duff layers and large logs. DC has a long-term response (about 50 days) to weather variations. The rating is defined in the range [0, +∞], with a default startup value of 15. In the CDS, this variable is called *drought_code*.

From these weather-driven fuel moisture calculations, the FWI model generates fire behavior indices in terms of:

- The **Initial Spread Index** (ISI), a measure of the rate at which a fire would spread in its early stages shortly after ignition. It combines the effects of wind and the FFMC on rate of spread without the influence of variable quantities of fuel. ISI is a numeric rating defined in the range [0, +∞]. In the CDS, this variable is called *initial_fire_spread_index*.
- The **Build Up Index** (BUI), the total amount of fuel available for combustion. It is a weighted combination of the DMC and DC and defined in the range [0, +∞]. In the CDS, this variable is called *build_up_index*.
  Lastly, the model computes the following two indices:
- The **Fire Weather Index** (FWI), a unit-less index of general fire intensity which results from the integration of ISI and BUI. It is defined in the range [0, +∞] and widely considered a general index of fire danger. In the CDS, this variable is called *fire_weather_index*.
- The **Daily Severity Rating** (DSR), a non-linear transformation of the Fire Weather Index which is intended to be directly proportional to the expected effort required for fire suppression and therefore widely used to predict the difficulty to control a fire. It is suitable to be averaged over space (i.e. region) and time (i.e. season). In the CDS, this variable is called *fire_daily_severity_rating*.

The above indices describe how fuel moisture and wind affect fire behavior if a fire is ignited^8^, but it does not make any assumption of fire ignition and its probability to strike. Fire danger, as conceptualized by the Fire Weather Index System is exclusively weather-driven, therefore it only quantifies a potential danger, not a real one as actual vegetation load, for example, is not taken into account.

In addition, start-stopping rules are not implemented and no mask for snow or precipitation is applied. This choice is mostly driven by the unavailability of good observations of snow cover and to give users the freedom to apply any masks of their choice. This also implies there is no *a-priori* overwintering applied as the decision on the length, start and stop of the fire season is left to the user. Figure 2 shows the mean of the determininstic high resolution (HRES) FWI values in 2017, while Fig. 3(a,b) show the mean and spread of the ensemble (ENS) FWI for the same period. Despite the difference in spatial resolution, the HRES and ENS means are overall similar. Both mean and spread of the ensemble seems to be highly spatially heterogeneous with smaller values in areas with relatively flat topography (e.g. east United States and Canada, Amazonian south America, north east Europe, north Russia) and larger values in mountainous (e.g. along the Rocky Mountains in the United States and Canada, along the Andes in south America, mountains surrounding the Meseta Central in Spain, East Africa, Ural Mountains in Russia, etc.) and desert areas. The latter areas would need to be masked out by the user due to the lack of vegetation load.

## Technical Validation

As FWI quantifies a potential danger, it is not an observable quantity but it can be validated against other existing reanalysis products. This section explores how FWI from GEFF-ERA5 compares to other reanalysis products, such as the Copernicus GEFF-ERAI and the Global Fire WEather Database (GFWED, GEOS-5 based) reanalysis developed by NASA’s Goddard Institute for Space Studies^20^. To allow a fair comparison, we only consider data points where GEFF-ERA5, GEFF-ERAI and GFWED are all non null. This is needed because, while GEFF-ERA5 and GEFF-ERAI have similar coverage (unchanged throughout the year), GFWED implements start-stopping rules which cause data coverage to change with time.

At each grid cell, errors are measured in terms of bias and anomaly correlation (Figs. 4 and 5), highlighting the effect of different model parametrisations and assumptions in various parts of the globe. Correlations are calculated using Pearson’s method and the related test is used to assess its statistical significance. Areas where correlations are not statistically significant are masked out. In Table 1 values are averaged over the macro-regions defined by the Global Fire Emission Database v4, which identify different fire regimes^21^. The average bias goes from −5 to +5, while the anomaly correlation ranges between 0.52 to 0.87.

**Table 1:** Summary table of the errors, in terms of bias and anomaly correlation, obtained when GEFF-ERA5 is compared to GEFF-ERAI and GFWED.

Region	Bias GFWED	Bias GEFF-ERAI	Anom. c. GFWED	Anom. c. GEFF-ERAI
Boreal North America	5.34	2.69	0.71	0.76
Temperate North America	4.76	1.13	0.77	0.80
Central America	0.97	4.59	0.81	0.80
North Hemisphere South America	1.23	2.17	0.62	0.66
South Hemisphere South America	−0.97	3.26	0.78	0.79
Europe	1.96	2.48	0.76	0.78
Middle East	−0.48	3.93	0.86	0.87
North Hemisphere Africa	1.01	3.87	0.85	0.82
South Hemisphere Africa	1.27	4.15	0.84	0.82
Boreal Asia	4.85	0.83	0.66	0.75
Central Asia	3.93	1.15	0.74	0.79
Southeast Asia	−5.43	3.51	0.81	0.84
Equatorial Asia	0.08	0.64	0.52	0.52
Australia and New Zealand	2.72	−0.84	0.83	0.87

Errors are averaged over homogeneous climatic regions.

Within each macro-regions, topography and local climate can affect differently model outputs. Figure 4a shows that, compared to its predecessor GEFF-ERAI, GEFF-ERA5 tends to provide similar fire danger estimates in areas with relatively flat topography while it shows higher biases (both positive and negative) in mountainous areas. This is not surprising given the substantial increase in resolution of ERA5 compared to ERAI which allows to better resolve orography providing a much improved localization of precipitation patterns, e.g. resolving the orographic up-wind and down-wind lifting of air^6^. The smaller anomaly correlations appear in places where the FWI is often close to zero such us the rainforests which experience intense precipitation all year around and the polar regions where temperatures are around zero for most of the year. Here low anomaly correlation values can be due to small numeric differences around zero (Fig. 5a).

When GEFF-ERA5 is compared to GFWED (Fig. 4b), areas in Asia and North America characterised by continental climates show the highest positive bias, while areas in humid subtropical regions in India and South America show the highest negative bias. The lowest anomaly correlations appear, again, in tropical and polar regions (Fig. 5b). Differences in the parameterization of local scale phenomena (e.g. convection) and the representation of larger scale dynamics (e.g. frontal systems) between the two atmospheric driving models can lead to different timing of both the diurnal cycle and the precipitation patterns. These are the main reasons responsible for the observed bias in these regions.

As just shown, in some areas there are substantial differences amongst the three model outputs which are the result of different parametrisations and spatial resolution. As the FWI is not a directly observable measure, it is not possible to establish which deterministic model output is closer to reality without introducing additional modelling uncertainties (e.g. calculating FWI at weather monitoring stations). Nevertheless, modelling uncertainties are to be acknowledged and treated explicitly. One way to do so is to use a composite of different models. Such a composite/ensemble model provides a range of model outputs rather than a deterministic value which is, theoretically, expected to encompass deterministic model estimates and reality itself. The ERA5 reanalysis database, in addition to its deterministic high resolution component, also provides a lower resolution 10 member ensemble. The ensemble is used to asses the spread of predictions often attributed to the natural variability^22^. The ensemble of ERA5 inputs is propagated through the GEFF model to produce an ensemble of fire danger indices, i.e. FWI ENS. Assuming a normal distribution at each grid cell, about 95% of the data values are expected to fall within two standard deviations of the mean value. Comparing the bias observed between GEFF-ERA5 and other reanalysis products (Fig. 4) with the spread (standard deviation) of the ensemble (Fig. 3) it appears there is enough variability in the ensemble model outputs to encompass the differences with the other reanalysis models for most areas on the globe. In regions where the spread is limited (tropical and cold to polar regions), there is no clear advantage in using the current ensemble in place of the high resolution reanalysis. The spread in FWI is, in the present system, only driven by the weather variability. The typical spread in the weather variables is less effective in producing spread in FWI when either the FWI is very low, typically below 10 or is very high, typically above 60–70. In these two ranges the FWI has an asymptotic behaviour and is less sensitive to the forcings. This might change with future releases of the dataset especially as one might think to perturb the parameters inside the GEFF model (e.g. drying time in the drought code of the FWI).

## Usage Notes

GEFF-ERA5 data, as any other dataset included in the Copernicus Climate Data Store is under the ECMWF Copernicus License version 1.2 (https://apps.ecmwf.int/datasets/licences/copernicus/). This is a full free and open data policy allowing anyone, anywhere in the world, to access and use the data.

Users might be interested in using these datasets for a variety of different purposes. As an example, one could explore the uncertainties associated with the fire danger estimates in a given location or more complex analysis like investigating the climatology of wildfire danger for a given region in relation to large scale meteorological phenomena like the El Niño Southern Oscillation (ENSO). In the two examples below we show these tasks are relatively straightforward to achieve using open source software tools such as *cdo*^23^ and the R package *caliver*^24^. The latter was specifically designed to efficiently manipulate ECMWF wildfire danger data.

### Illustration of use: fire danger trend in June in Pedrógão Grande in Portugal

Compared to the previous reanalysis dataset, ERA5 provides a set of ensemble simulations that can be used to asses the expected variability of the predictions. An example application of these dataset is provided here, looking at the catastrophic forest fire occurred in the Pedrógão Grande area, Leiria region (Portugal) in June 2017 which caused 66 casualties and triggered structural changes in the way assistance of fire events are addressed by the European Commission (https://ec.europa.eu/commission/news/resceu-2018-dec-12_en). The fire is believed to have been caused by a dry thunderstorm and has spread very quickly due to a concurrent heatwave affecting the region with temperatures of more than 40C, which are very unusual so early in the summer season. The event triggered a vast debate on how fire regimes are changing with climate and human activities on the landscape, also showing how emissions from mega-fires have intensified in magnitude and duration globally^25^.

Using FWI as a proxy for flammability, it is possible to construct a time series as a concatenation of June days using the entire record of ERA5 reanalysis for the gridbox including the Pedrógão Grande location, to understand if there is a clear change in fire danger in the area in the past 40 years. The daily time series was decomposed into a linear trend, seasonal and random component. The trend analysis highlighted a slight increase of FWI over the past 40 years, going from an average of 13 to 17. The direction of the trend is shown in Fig. 6 as a red line, starting from the origin to highlight the differences (rather than the absolute values) between the beginning and end of the period of interest. An interesting aspect is provided by comparing the linear trend with the spread of the ensemble defined as the difference between the maximum and the minimum simulated values across the 10 members. The monthly average spread of the ensemble is shown as gray bars in Fig. 6 and it ranges between 2 and 10, depending on the year and the intrinsic predictability of the atmospheric conditions in June. Although reanalysis is often used as a proxy for observations, it is ultimately the output of a model. Using the ensemble reanalysis product, in place of the deterministic one, allows to quantify the uncertainties associated with a given estimate and convey a measure of confidence in the model output. In this specific analysis the uncertainty in the prediction, determined by the ensemble spread, encompasses the trend rise in FWI suggesting that the trend is not particularly significant. This very simple decomposition leads to a substantial contribution provided by the random component suggesting that, for this analysis, a linear trend might not be the right modelling choice. Further investigations are beyond the scope of this section, as it is simply intended to illustrate possible avenues for data usage.

### Illustration of use: El Niño driven fire danger in South East Asia

The ENSO is a naturally occurring anomalous state of tropical Pacific coupled ocean-atmosphere conditions and is a primary predictor for global climate disruptions. The establishment of a positive or negative ENSO are usually monitored using a Multivariate index (MVI) obtained by extracting the leading combined Empirical Orthogonal Function (EOF) of five different variables over the tropical Pacific basin (30°S–30°N and 100°E–70°W)^26,27^.

There have been a number of studies showing how positive ENSO can establish favourable conditions for the triggering and sustainability of wildfires in several areas around the world^28^. One of the most affected regions is Indonesia^29,30^ where human-induced fires take place every year for the purpose of clearing land and removing agricultural residues. During intense dry seasons these fires can penetrate into degraded subsurface peat soil with enhanced flammability. They are extremely difficult to extinguish and can burn continuously until the return of the monsoon rains, usually in late October or early November. The strength and prevalence of these fires are strongly influenced by large-scale climate patterns like ENSO when dry conditions are well above the average^31,32^. The intensification of fire danger persistence is clearly shown looking at the number of days in a given year in which the FWI is above the 98^th^ percentile of its climatology. The boxplots in Fig. 7 show that the median in the period 1997–1998 (which was characterised by a very strong positive ENSO, see also Fig. 8a) is around 13 days with some locations reaching up to 60 days above the 98^th^ percentile. In comparison, in 2010 when a very strong negative ENSO was in place (Fig. 8b), the number of days above threshold was well below 20 in most of South East Asia. It is, therefore, evident how years characterised by very strong positive ENSO tend to have a very prolonged period characterised by extreme fire danger.

To summarise, the teleconnection between ENSO and South East Asia dry anomalies has been vastly documented in the literature and can be easily identified in the presented dataset. Similar analysis could be conducted for other regions and other fire danger variables.

## Data Availability

The fire indices have been generated using the open source model GEFF v3.1 (https://git.ecmwf.int/projects/CEMSF/repos/geff). The code to reproduce the results of this manuscript is openly available on a public repository on GitHub (https://github.com/cvitolo/paper_geff_era5).
